# Association of serum vitamin D concentration with the final course of hospitalization in patients with COVID-19

**DOI:** 10.3389/fimmu.2023.1231813

**Published:** 2023-09-01

**Authors:** Klaudia Konikowska, Katarzyna Kiliś-Pstrusińska, Agnieszka Matera-Witkiewicz, Krzysztof Kujawa, Barbara Adamik, Adrian Doroszko, Krzysztof Kaliszewski, Michał Pomorski, Marcin Protasiewicz, Janusz Sokołowski, Katarzyna Madziarska, Ewa Anita Jankowska

**Affiliations:** ^1^ Department of Dietetics and Bromatology, Wroclaw Medical University, Wroclaw, Poland; ^2^ Clinical Department of Paediatric Nephrology, Wroclaw Medical University, Wroclaw, Poland; ^3^ Screening of Biological Activity Assays and Collection of Biological Material Laboratory, Wroclaw Medical University Biobank, Wroclaw Medical University, Wroclaw, Poland; ^4^ Statistical Analysis Centre, Wroclaw Medical University, Wroclaw, Poland; ^5^ Clinical Department of Anaesthesiology and Intensive Therapy, Wroclaw Medical University, Wroclaw, Poland; ^6^ Clinical Department of Internal and Occupational Diseases, Hypertension and Clinical Oncology, Wroclaw Medical University, Wroclaw, Poland; ^7^ Clinical Department of General, Minimally Invasive and Endocrine Surgery, Wroclaw Medical University, Wroclaw, Poland; ^8^ Clinical Department of Gynecology and Obstetrics, Wroclaw Medical University, Wroclaw, Poland; ^9^ Institute of Heart Diseases, Wroclaw Medical University, Wroclaw, Poland; ^10^ Clinical Department of Emergency Medicine, Wroclaw Medical University, Wroclaw, Poland; ^11^ Clinical Department of Nephrology and Transplantation Medicine, Wroclaw Medical University, Wroclaw, Poland; ^12^ Institute of Heart Diseases, University Hospital, Wroclaw, Poland

**Keywords:** SARS-CoV-2, vitamin D, COVID-19, mortality, vitamin D deficiency, public health

## Abstract

**Background:**

Vitamin D deficiency is a substantial public health problem. The present study evaluated the association between vitamin D concentration and hospitalization and mortality risk in patients with coronavirus disease 19 (COVID-19).

**Methods:**

This study used the COronavirus in LOwer Silesia (COLOS) dataset collected between February 2020 and June 2021. The medical records of 474 patients with confirmed severe acute respiratory syndrome 2 (SARS-CoV-2) infection, and whose vitamin D concentration was measured, were analyzed.

**Results:**

We determined a significant difference in vitamin D concentration between discharged patients and those who died during hospitalization (p = 0.0096). We also found an effect of vitamin D concentration on the risk of death in patients hospitalized due to COVID-19. As vitamin D concentration increased, the odds ratio (OR) for death slightly decreased (OR = 0.978; 95% confidence interval [CI] = 0.540-0.669). The vitamin D concentration cutoff point was 15.40 ng/ml. In addition, patients with COVID-19 and serum 25-hydroxyvitamin D (25(OH)D) concentrations < 30 ng/ml had a lower survival rate than those with serum 25(OH)D ≥ 30 ng/ml (log-rank test p = 0.0018). Moreover, a Cox regression model showed that patients with an estimated glomerular filtration rate (eGFR) ≥ 60 ml/min/1.73 m^2^ and higher vitamin D concentrations had a 2.8% reduced risk of mortality (hazard ratio HR = 0.972; CI = 0.95-0,99; p = 0.0097).

**Conclusions:**

The results indicate an association between 25(OH)D levels in patients with COVID-19 and the final course of hospitalization and risk of death.

## 1 Introduction

Vitamin D deficiency is a global problem that affects healthy and sick people (1). Race is a significant risk factor for vitamin D deficiency (2). The overall prevalence rate of vitamin D deficiency in the U.S. population was 41.6%, with the highest rate among African Americans (82.1%), followed by Hispanics (69.2%), and lowest among non-Hispanic Whites (30.9%) (3). The risk group consists mainly of overweight and elderly individuals who require higher doses of vitamin D (4, 5). Vitamin D deficiency is also often detected in patients with liver and kidney disorders (4, 6–8). Health benefits of vitamin D supplementation and sunlight exposure include reduced risk of many chronic diseases, including autoimmunity, cardiovascular disease, infections, and neurocognitive dysfunctions (1). Adequate vitamin D levels also prevent frailty and fractures (9).

Studies show that vitamin D has pleiotropic effects (10, 11). Calcitriol, 1,25-dihydroxycholecalciferol [1,25(OH)2D], is the active form of vitamin D and participates in numerous physiological processes while exhibiting immunomodulatory effects (12). It promotes immune cell proliferation and differentiation, modulates lymphocyte activity, and reduces pro-inflammatory cytokine (tumor necrosis factor alpha [TNF-α] and interleukin-1 [IL-1]) concentration while increasing anti-inflammatory cytokine (IL-4, IL-5, and IL-10) concentration (4). In addition, the action of 1,25(OH)2D is mediated by a highly specific, intracellular vitamin D receptor (VDR) (4, 13). As a nuclear receptor, ligand activation of the VDR leads to protein binding to specific sites in the genome, resulting in modulation of target gene expression (14). The pleiotropic effect of 1,25(OH)2D is believed to be active in the presence of VDR and 25(OH)D-1α-hydroxylase (CYP27B1) in many tissues, which allows the extrarenal synthesis of the active 1,25(OH)2D. The participation of 25(OH)D in the endocrine, autocrine, and paracrine pathways seems to be crucial for reducing the risk of cancer development, autoimmune diseases (e.g., multiple sclerosis, type 1 diabetes, and bronchial asthma), cardiovascular diseases, strokes, type 2 diabetes, and neurocognitive disorders (13, 15). 25(OH)D also reduced the incidence of recurrent infections (2, 16).

Vitamin D deficiency is linked to increased autoimmunity and higher susceptibility to respiratory tract infection (RTI) (17, 18). Nevertheless, the association between antimicrobial, antiviral, and anti‐inflammatory effects may reduce the risk of asthma exacerbation, often caused by RTI and characterized by dysregulated pulmonary inflammation (19, 20). In addition, other studies have demonstrated an association between vitamin D deficiency and higher intensive care unit (ICU) mortality rates (21, 22).

The therapeutic role of vitamin D in patients with coronavirus disease 2019 (COVID-19) is currently a subject of discussion (23). Low vitamin D concentration has been linked to an increased predisposition to severe acute respiratory syndrome coronavirus 2 (SARS-CoV-2) infection (24) and a severe course of COVID-19 (25). Furthermore, it has been hypothesized that vitamin D may prevent and/or alleviate SARS-CoV-2 infection (23). However, the results of such research are not conclusive.

Based on studies conducted in 20 countries in Europe, Illie et al. (26) demonstrated a significant negative correlation between total 25-hydroxyvitamin D [25(OH)D] serum concentration and death in SARS-CoV-2 patients. In contrast, Orchard et al. (27) reported a negligible difference in vitamin D levels among hospitalized and ICU patients with COVID-19. Moreover, a non-significant difference in the clinical course was found between patients with low and normal vitamin D concentrations (27). Similarly, no beneficial effect of vitamin D3 treatment was found in patients with COVID-19 by Marani et al. (28). Administration of a large single dose of vitamin D3 (500,000 IU) did not prevent respiratory function deterioration in patients hospitalized with mild to moderate COVID-19, and there was no significant effect on the length of hospital stay (28).

Our study is one of the first to look at the association of vitamin D with adult mortality in the Polish population. Based on current knowledge, there are only a few published studies from Poland on vitamin concentrations in patients with COVID-19 (29, 30). In the study by Ziuzia-Janiszewska et al. (29), potential predictors of a severe course of COVID-19 in young adults (20-45 years old) were identified, including low vitamin concentration as one of the predictors. In this study, vitamin D concentration was tested in 81 out of 229 patients with SARS-CoV-2. Ziuzia-Janiszewska et al. (29) found that, among others, obesity, comorbidities, higher levels of CRP, IL-6, creatinine, urea, and also lower eGFR value, albumin, calcium, and vitamin D concentration may be associated with poor COVID-19 outcomes. In another study from Poland, vitamin D concentrations were analyzed in 45 out of 52 pregnant women with confirmed SARS-CoV infection (30). It was shown that approximately 62% of pregnant women with COVID-19 had a decreased concentration of vitamin D (mean value – 27.15 ng/ml) (30).

Our study evaluated the association between vitamin D concentration and the final course of hospitalization and risk of death in patients treated for COVID-19. COVID-19 can affect many organs of the human body, including kidneys (31). The mechanism of kidney involvement in COVID-19 appears to be multifactorial. Thus far, data suggest effects of direct viral infection (viral tropism to the renal system), hypoxia, inflammatory syndrome-mediated injury, hemodynamic instability, vascular injury, and hypercoagulable state (32–35). In our study, an accurate analysis of the relationship between vitamin D concentration and baseline estimated glomerular filtration rate (eGFR) and the probability of a patient’s survival outcome up to 90 days after hospital admission was performed. In addition, the association between vitamin D and other predictors of the incidence of death up to 90 days after COVID-19 hospitalization was examined using a Cox regression model. We hypothesized that low 25(OH)D plasma levels can predict poorer survival outcomes. We also aimed to evaluate whether 25(OH)D plasma levels predict mortality in adults with COVID-19 while considering potential confounders.

## 2 Methods

### 2.1 Study design and participants

Our research was part of the COronavirus in LOwer Silesia (COLOS) study of patients with laboratory-confirmed SARS-CoV-2 infection. The medical records of 474 patients with vitamin D concentration measurements were analyzed retrospectively. The patients were hospitalized at the University Hospital in Wroclaw between February 2020 and June 2021.

SARS-CoV-2 infection was confirmed by positive reverse transcriptase-polymerase chain reaction (RT-PCR) for viral ribonucleic acid (RNA) from a nasopharyngeal swab.

The Institutional Review Board and Bioethics Committee of Wroclaw Medical University approved this COLOS study (No: KB-444/2021). The data were collected retrospectively, and written informed consent to participate in the study was not required. The Bioethics Committee approved the publication of anonymized data.

### 2.2 Study procedures

The demographic data and information on medical history, previous medication, symptoms at admission, laboratory tests, and in-hospital clinical courses were derived from electronic hospital medical records. The presence of comorbidities and information about smoking was established from an interview with the patient on admission.

Laboratory assessment measured vitamin D status, IL-6, C-reactive protein (CRP), and renal function tests, including creatinine, were measured and eGFR values were calculated. Different indexes are used for kidney function assessment and the eGFR remains one of the best markers (36). eGFR was calculated based on the Modification of Diet in Renal Disease Study equation (36). A chemiluminescence method (Alinity i 25-OH Vitamin D Reagent Kit, Abbott, USA) evaluated the total serum 25(OH)D concentration.

Surviving patients were followed up by telephone after 3 months. Information was obtained directly from patients, their relatives, or the hospital system. Government General Registry Office data on death were used for the study.

### 2.3 Statistical analysis

Continuous data were expressed as mean and standard deviation (SD) when a normal distribution occurred. In the absence of a normal distribution, medians, and interquartile ranges were reported. The categorical data were presented as frequencies and percentages. Given the sample size in the study, the continuous variable was analyzed using parametric tests. Although parametric tests are designed for normal data distribution in general, it has been shown recently that the ANOVA is robust to the violation of this assumption (37, 38). It matters especially when the sample size is large compared to a threshold sample size (N = 30), which has been proposed as a criterion for reliably using parametric tests (when the assumption of the variance homogeneity is met) (39), with a sample size being considered as “large” when it contains hundreds of data (37, 38). Categorical variables were compared using Pearson’s Chi-squared or Fisher’s exact tests when the sample size was smaller than five.

ANOVA with Welch correction evaluated the relationship between the vitamin D concentration and the final hospitalization course. As the variance was unequal among the compared group (Levene’s test: F_3, 470 = _3.18, p = 0.024), Tamhane’s T2 *post hoc* test assessed differences between the groups. The final course of hospitalization was described as a categorical variable and divided into four stages: 0 - completed with discharge; 1 - completed transfer from the University Clinical Hospital to another acute hospital for specialized treatment due to new problems/deterioration of the patient’s condition; 2 - completed by transfer to another hospital outside of the University Clinical Hospital for rehabilitation or docking of patients who could not be discharged home; 3 - fatal.

The effect of vitamin D concentration on the risk of death during hospitalization was assessed using a receiver operating characteristic (ROC) curve. The area under the curve (AUC) and confidence intervals (CIs) were calculated. The analysis established a Youden index and estimated a cutoff point for vitamin D concentration.

A Cox model evaluated the relationship between vitamin D concentration and eGFR values and the probability of patients’ survival up to 90 days from the start of hospitalization. The results of patients’ survival probabilities were presented using Breslow survival curves, as were patients’ survival probabilities concerning vitamin D concentration and eGFR value. According to the US National Kidney Foundation, chronic kidney disease (CKD) can be defined as an eGFR < 60 ml/min/1.73 m^2^ (40). We assumed that the expected normal clinical range of eGFR is ≥ 60 ml/min/1.73 m^2^. To visualize the effect of vitamin D concentration not disturbed by the effect of eGFR, the latter was assumed to be equal to 76.2 ml/min/1.72 m^2^ (the arithmetic mean).

As the effects of the vitamin D concentration were statistically significant in the Cox regression, in the next step, Kaplan-Meier analysis with log-rank test compared the survival of patients up to the 90th day after hospital admission, divided into two groups based on the vitamin D concentration determined at the beginning of hospitalization. Patients were grouped into two serum 25(OH)D level categories: < 30 ng/ml and ≥30 ng/ml. Vitamin D sufficiency was defined as a blood level of ≥ 30 ng/ml. Vitamin D levels < 30 ng/ml were considered low.

In the last stage of the analysis, the hazard ratio (HR) and 95% CI of mortality (the incidence of death in patients with COVID-19 up to 90 days from the start of hospitalization) in relation to vitamin D concentration (as a continuous variable) and some other co-variables that are considered potentially important predictors were estimated using Cox proportional hazard regression models. The other variables entered into the model were sex, age at admission, CRP level, and the presence of heart disease. Because eGFR is usually considered an important predictor when assessing COVID-19 severity, a Cox model was used for the two patient groups separately. Patients with a measured vitamin D concentration were divided into two groups according to eGFR at admission: eGFR ≥ 60 ml/min/1.73 m^2^ or eGFR < 60 ml/min/1.73 m^2^.

In all the Cox regression models, p was > 0.2 for all the predictors in the test of proportional hazard assumptions. The assumption of a linear form of continuous covariates was assessed by plotting the Martingale residuals against continuous covariates. Goodness-of-fit of the Cox regression model was assessed using the statistics “concordance” (see below).

A p-value < 0.05 was considered statistically significant. Statistical analyses employed Statistica v.13.3 software (TIBCO Software Inc., Krakow, Poland) (descriptive statistics, Levene’s test, proportional hazard test, and preparing scatterplots of Martingale residuals), SPSS 28.0 (Welch’s ANOVA), and R-packages “survival” (41) and “survminer” (42) (Cox regression, forest plot preparation, Kaplan-Meier curves, and log-rank test “concordance”).

## 3 Results

Vitamin D concentration in the blood of 474 patients hospitalized for SARS-CoV-2 infection was evaluated. The mean age of patients was 63 (± 15.3) years, and 276 (58.1%) patients were men. Comorbidities such as diabetes, hypertension, cardiovascular disease, and chronic kidney disease were present in 128 (26.9%), 259 (54.5%), 124 (26.1%), and 48 (10.1%) patients, respectively (**Table 1**).

**Table 1:** Patients’ characteristics and clinical outcomes.

Variables	
Age, mean (SD), years	63.0 (15.3)
Men, No. (%)	276 (58.1)
Comorbidities occurrence*	
Diabetes, No. (%)	128 (26.9%)
Hypertension, No. (%)	259 (54.5%)
Cardiovascular disease, No. (%)	124 (26.1%)
Chronic kidney disease, No. (%)	48 (10.1%)
Tumor, No. (%)	29 (6.1%)
Smoking, No. (%)*	
Never	421 (88.6%)
Former	32 (6.7%)
Current	21 (4.4%)
Hospitalization course	
The number of days in hospital, mean (SD), days	20.3 (15.5)
Transferring the patient to ICU, No. (%)	103 (21.7%)
Tracheostomy, No. (%)	47 (9.9%)
Intubation during hospitalization, No. (%)	98 (20.6%)
Shock during hospitalization, No. (%)	89 (18.7%)
Deterioration of the patient’s condition during hospital stay, No. (%)	172 (36.2%)
Initiation of renal replacement therapy, No. (%)	41 (8.6%)
SIRS, No. (%)	65 (13.7%)
Death in hospital, No. (%)	96 (20.2%)
Laboratory values	
25(OH)D, ng/ml (Me, IQR)	20.55 (20.30)
Creatinine, mg/dl (Me, IQR)	0.95 (0.44)
CRP, mg/l (Me, IQR)	61.18 (101.29)
IL-6, pg/mL (Me, IQR)	21.85 (43.15)

* data from the interview with the patient on admission; SD, standard deviation; No., number; ICU, Intensive Care Unit; SIRS, systematic inflammatory response syndrome; 25(OH)D, 25-hydroxyvitamin D; CRP, C-reactive protein, IL-6, Interleukin-6, Me, median; IQR, interquartile range.

The relationship between vitamin D concentration and the final hospitalization course was statistically significant (ANOVA: F_3, 470 = _4.82, p = 0.003) (**Table 2**). A statistically significant difference in vitamin D concentration compared to discharged patients and those who were transferred urgently to another hospital due to new health problems/deterioration of their condition was determined. Moreover, discharged patients had significantly higher mean vitamin D concentrations than patients who died during hospitalization. In addition, patients who were transferred urgently to another hospital due to new health problems/deterioration of their condition had significantly lower mean vitamin D concentrations than patients transferred to another hospital for rehabilitation or docking.

**Table 2:** Vitamin D concentration and the course of hospitalization (ANOVA test, p-values for post-hoc Tamhane’s T2 test).

		n (%)	mean (95% CI)	Me			Pairwise post-hoc comparisons			
							0	1	2	3
Hospitalization *	0	302 (63.6%)	26.0 (23.9-28.1)	23.1	Hospitalization *	0	–	0.0002	0.5503	0.0096
	1	21 (4.4%)	16.0 (12.2-19.8)	13.4		1	0.0002	–	0.0362	0.4434
	2	55 (11.6%)	23.0 (19.7-26.3)	22.0		2	0.5503	0.0362	–	0.7251
	3	96 (20.2%)	20.1 (17.0-23.1)	14.9		3	0.0096	0.4434	0.7251	–

Hospitalization*: 0 - completed with discharge; 1 - completed transfer from the University Clinical Hospital to another acute hospital for specialized treatment due to new problems/deterioration of the patient’s condition; 2 - completed by transfer to another hospital outside the University Clinical Hospital for rehabilitation or docking of patients who cannot be discharged home; 3 – fatal; CI, confidence interval; Me, median.

**Figure 1:**
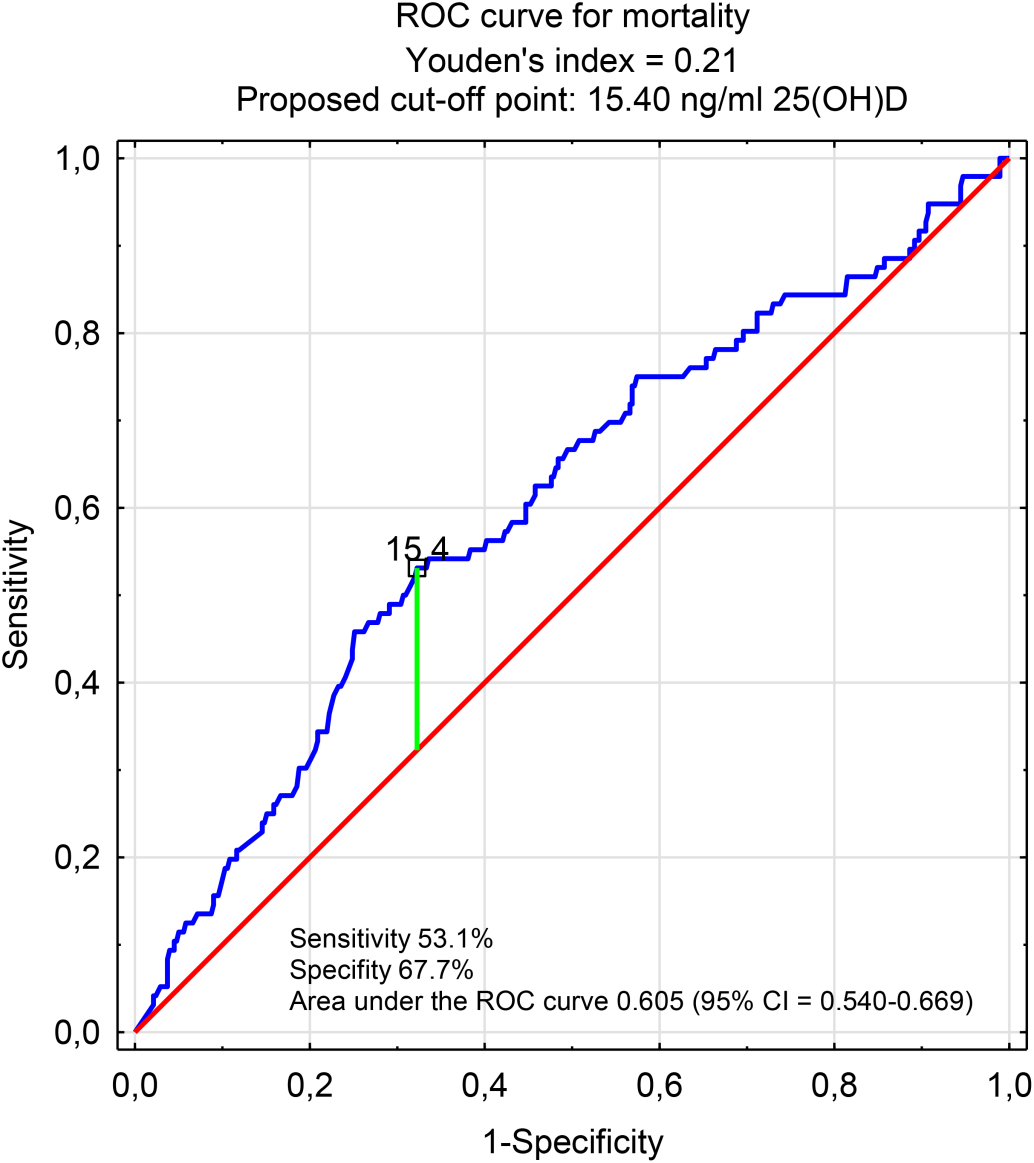
Area under the ROC curve of vitamin D serum level for in-hospital mortality of patients with COVID-19.

In our study, the effect of vitamin D concentration on death risk due to COVID hospitalization was also analyzed. **Figure 1** shows the area under the ROC curve of vitamin D serum level for in-hospital mortality of patients with COVID-19. The AUC of the 25(OH)D ROC curve was 0.605 (CI 95% = 0.540-0.669; p = 0.0106) and shows that as vitamin D concentration increased, the odds ratio (OR) for death slightly decreased (OR = 0.978). Without adjusting for confounders, the sensitivity and specificity of vitamin D concentration as a single predictor for mortality in patients hospitalized for COVID-19 were poor (AUC = 0.605; 95% CI = 0.540-0.669). The cutoff point for vitamin D concentration was 15.40 ng/ml.

The study also assessed the probability of survival up to 90 days after the onset of hospitalization for SARS-CoV-2 infection, taking into account vitamin D concentration and eGFR values during hospital admission. Using Cox regression, we demonstrated a statistically significant effect of vitamin D concentration and baseline eGFR on the probability of survival (vitamin D: HR = 0.98; 95% CI = 0.969-0.995; p = 0.0075; eGFR value: HR = 0.99; 95% CI = 0.981-0.993; p = 0.000009, model concordance - 0.631).

**Figure 2:**
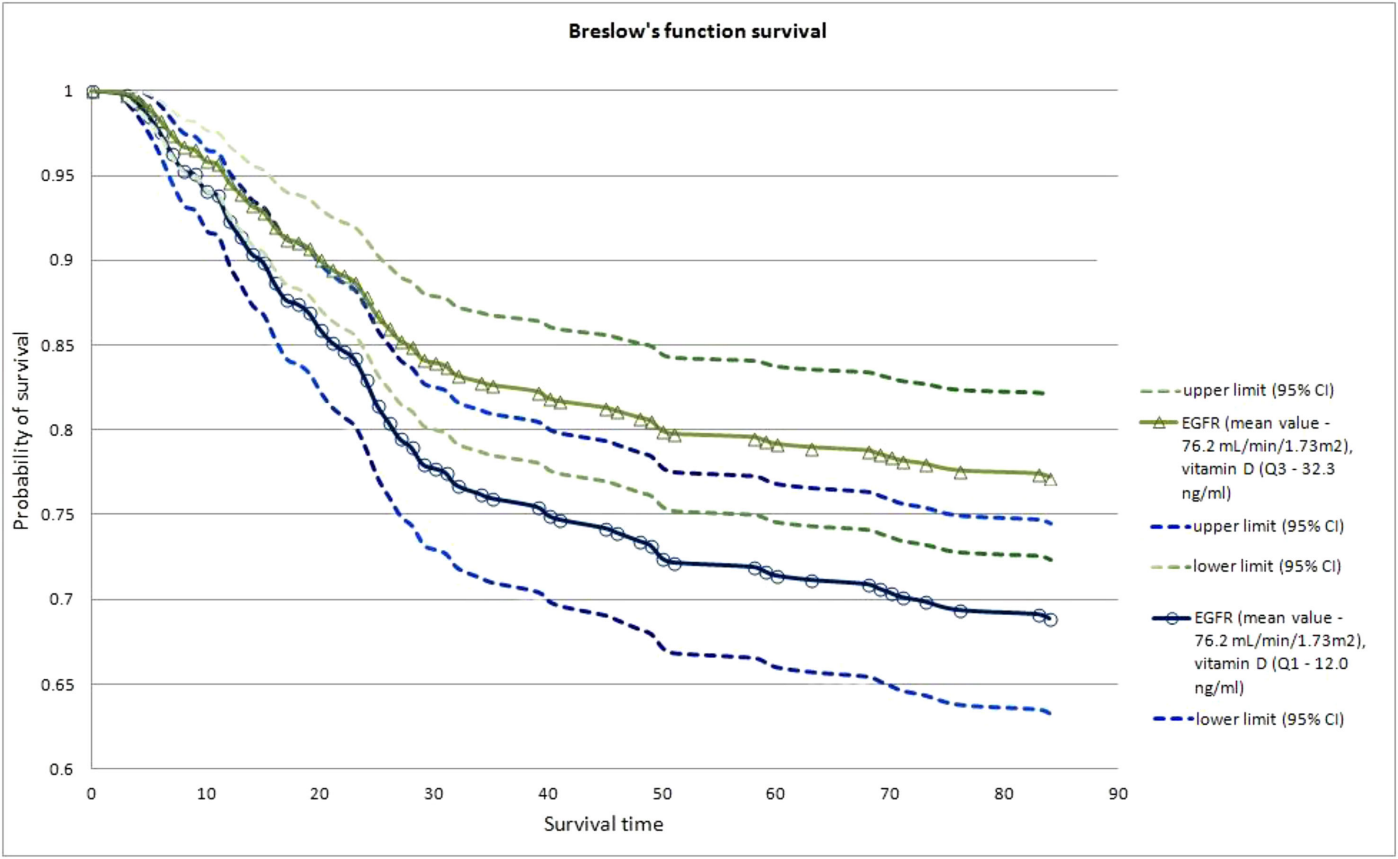
Breslow survival curves for patients with COVID-19 up to 90 days after hospitalization in relation to low (1^st^ quartile Q1,) and high (3^rd^ quartile Q3) vitamin D level, at constant eGFR level, equal to 76.2 mL/min/1.73m^2^ (arithmetic mean).

To illustrate the effect of vitamin D concentration on survival probability, Breslow survival curves were prepared for two vitamin D concentrations to include the first and third quartiles. Survival probability at a low vitamin D concentration was markedly lower when compared to the high vitamin D concentration quartile (68.9% vs. 77.2%) at the same eGFR value (see “Statistical analysis” section and **Figure 2** for details).

**Figure 3:**
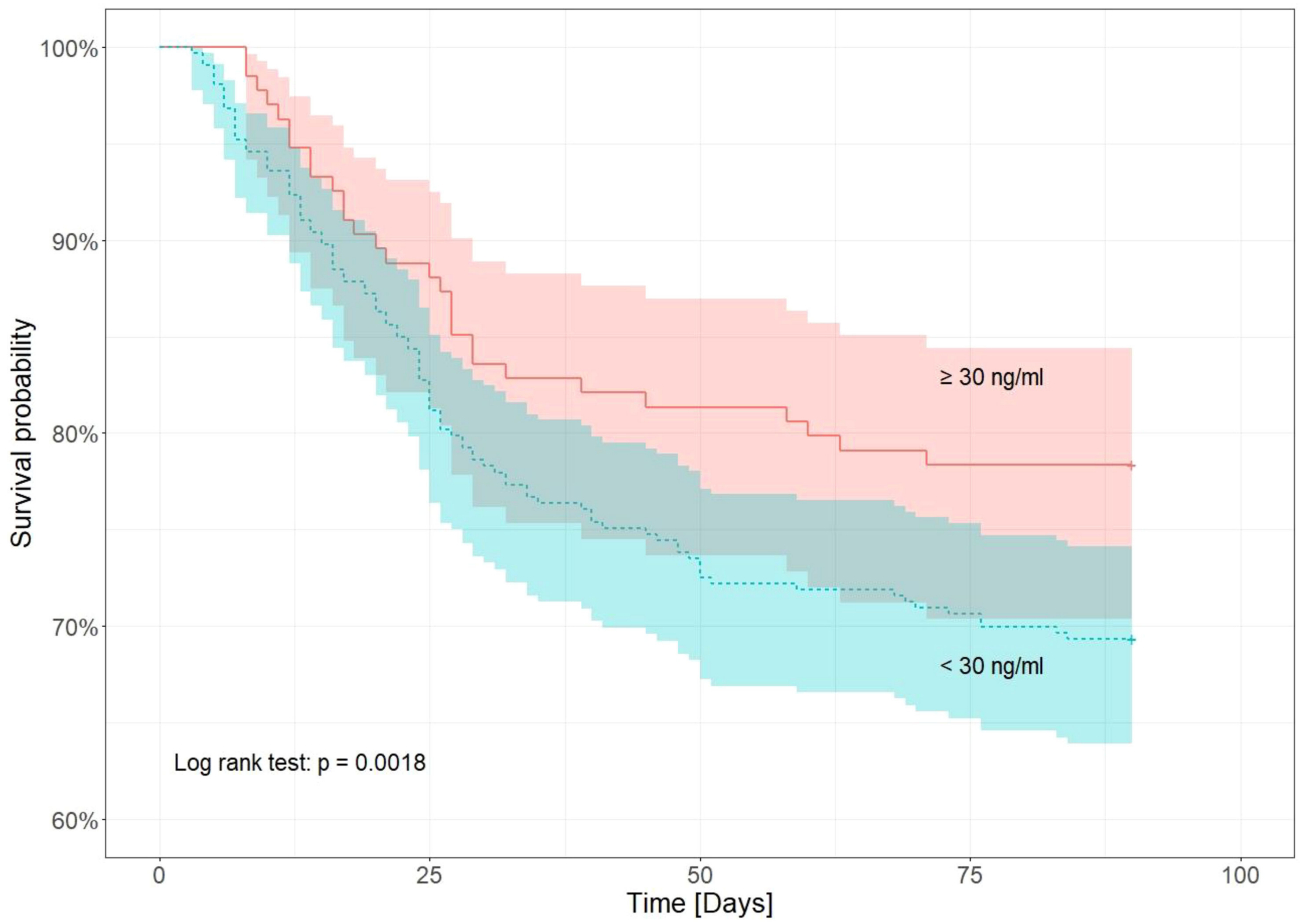
Kaplan–Meier survival analysis of COVID-19 patients until the 90th day from admission to hospital in relation to vitamin D concentration (<30 ng/ml and ≥ 30 ng/ml).

**Figure 3** shows Kaplan-Meier curves for the survival probability of patients with COVID-19 up to 90-days after admission to the hospital according to vitamin D status. Patients with COVID-19 and serum 25(OH)D concentrations < 30 ng/ml presented a lower survival rate than those with serum 25(OH)D ≥ 30 ng/ml (log-rank test p = 0.0018).

**Figure 4:**
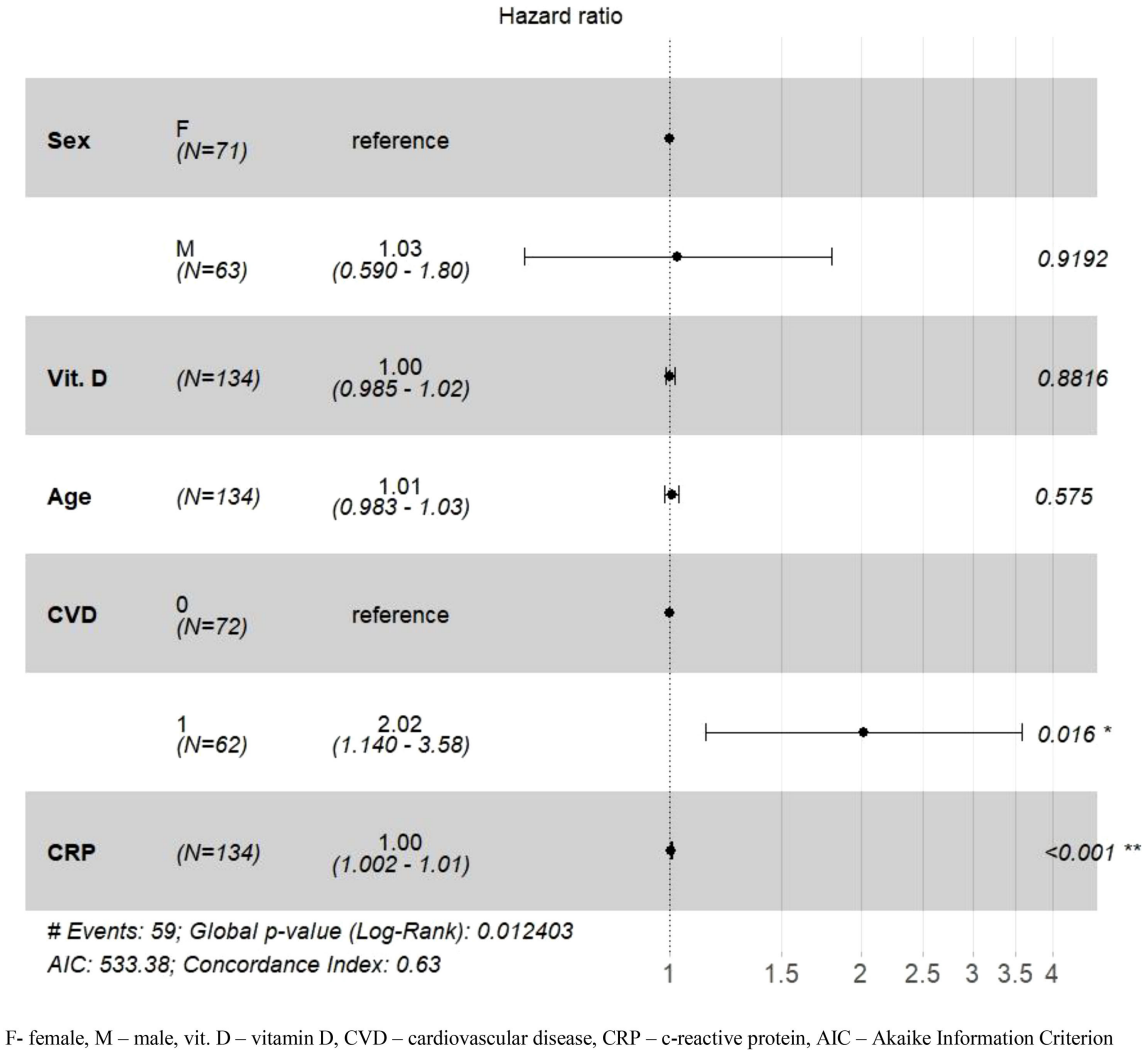
Adjusted hazard ratios (HR) for 90-day mortality in the group of patients with eGFR < 60 ml/min/1.73 m^2^. *p-value <0.05; **p-value <0.01.

**Figure 5:**
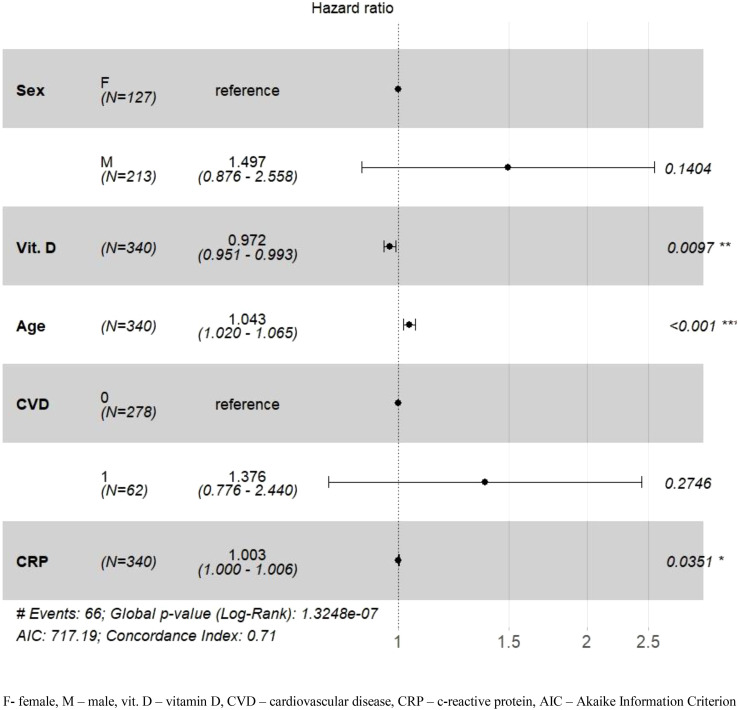
Adjusted hazard ratios (HR) for 90-day mortality in the group of patients with eGFR ≥ 60 ml/min/1.73 m^2^. *p-value <0.05; **p-value <0.01; ***p-value <0.001.

The Cox regression model showed that the association between vitamin D concentration and mortality risk in patients with COVID-19 depended on baseline eGFR (**Figures 4**, **5**). In patients with COVID-19, it relied on baseline eGFR (**Figures 4**, **5**), though in those with eGFR < 60 ml/min/1.73m^2^, vitamin D concentration was not significantly associated with mortality risk (p = 0.882). In contrast, patients with an eGFR ≥ 60 ml/min/1.73 m^2^ and higher vitamin D concentrations had a 2.8% reduced mortality risk (HR = 0.972; CI = 0.95-0.99; p = 0.0097) even after adjusting for potential confounders such as sex, heart disease, and CRP value. The mortality risk also increased by 4% with age in COVID-19 patients with eGFR values ≥ 60 ml/min/1.73 m2. Model concordance for the patient groups amounted to 0.63 for patients with eGFR < 60 ml/min/1.73 m^2^ and 0.71 for patients with an eGFR ≥ 60 ml/min/1.73 m^2^.

## 4 Discussion

This study presented the results of the association between vitamin D concentrations and the risk of death in patients hospitalized with COVID-19. Campi et al. (43) evaluated the vitamin D concentrations of 103 patients treated for severe COVID-19 (study group) and compared them to 52 patients with mild COVID-19 and 206 patients without confirmed SARS-CoV-2 infection (control group) and found that 25(OH)D concentrations were lower in COVID-19 patients who died in hospital (13.2 ± 6.4 ng/ml) compared to patients who survived (19.3 ± 12.0 ng/ml; p=0.0003). Moreover, they discovered an inverse correlation between 25(OH)D and mortality regardless of age, sex, diabetes, platelet count, and levels of IL-6, CRP, lactate dehydrogenase (LDH), neutrophils, and lymphocytes. The study found that a 1 ng/ml 25(OH)D increase was associated with a 4% reduction in the risk of death from COVID-19 (43).

In the current study, vitamin 25(OH)D values relevant to survival were in analogous convergence. We found that patients with COVID-19 and vitamin D values of 15.40 ng/ml or less at admission had a higher risk of death during hospitalization than patients whose vitamin D concentrations were above 15.40 ng/ml. Hafez et al. (44) reported similar findings. This study showed a statistically significant correlation between 25(OH)D deficiency (<12 ng/ml) and in-hospital mortality (p = 0.04). In an unadjusted logistic regression model, the probability of mortality increased significantly among patients with serum 25(OH)D concentration <12 ng/ml compared to those with a higher serum concentration (OR = 7.86, 95% CI =1.43–37.49). In addition, Hafez et al. (44) used an adjusted logistic regression model for sex and age (Model 1), with a cutoff point of 25(OH)D < 12 ng/ml and 25(OH)D < 20 ng/ml adjusted for sex, age, race, and comorbidities in multivariate logistic regression in Models 2 and 3, respectively. The authors revealed that a serum 25(OH)D concentration <12 ng/ml significantly increased mortality 12-fold in Model 1 and 62-fold in Model 2.

In our study, the Cox regression model showed that the mortality risk also increased by 4% with age in COVID-19 patients with eGFR values ≥ 60 ml/min/1.73 m^2^. It is worth mentioning that the value of eGFR decreases with age by approximately 1 ml/min/m^2^ per year starting from the third decade of life (45). In addition, renal hyperfiltration is associated with a higher risk of cardiovascular disease and all-cause mortality (46, 47). Hafez et al. (44) also showed increased mortality of 13% for each 1-year increase in age. Researchers suggest that immune system functions decline with increasing age, which may be one of the risk factors for death (44).

Another study involving 464 patients with COVID-19 found that 25(OH)D concentrations < 12 ng/ml were significantly associated with a higher risk of severe SARS-CoV-2 infection and mortality (48). Al Safar et al. (48) determined predictors of mortality using binary regression analysis with adjusted and unadjusted models. Age was significantly associated with the risk of death regardless of the model used. In addition, a significant predictor of death in the adjusted models was serum vitamin D concentration < 12 ng/mL, which was associated with a 2.55-fold increased risk of death after adjusting for sex and age, and a 2.58-fold increased risk of death after adjusting for sex, age, and comorbidities (48). Comorbidities such as heart disease, diabetes, kidney disease, and metabolic disease only manifested as risk factors for death in unadjusted models (48). In our study, heart diseases were also a significant risk factor for death, but only for patients with an eGFR < 60 ml/min/1.73 m^2^.

In our study, Cox regression found no association between sex and the risk of death up to 90 days from hospitalization for COVID-19. In the Al Safar et al. (48) study, as in our study, sex was not a significant predictor of death in patients with COVID-19. Evaluating the association between sex and 25(OH)D with risk of death requires further studies in a homogeneous group of patients.

The current study showed a significant effect of eGFR values and vitamin D concentration on survival probability up to 90 days after hospitalization, with a proportionally higher survival probability and vitamin D concentration observed when comparing the same baseline eGFR values (**Figure 2**). Thus, it may be suggested that higher 25(OH)D concentrations are important for a better prognosis in patients with COVID-19. On the other hand, the Cox regression model showed that, while analyzing additional variables such as age, gender, heart disease, and CRP value, the association between vitamin D concentration and mortality risk in patients with COVID-19 depended on baseline eGFR. Kidneys are the main site for the conversion of 25-hydroxyvitamin D into circulating calcitriol, and are essential for the health benefits of endocrine VDR activation (49). Vitamin D deficiency increases progressively in the course of kidney disease and is associated with accelerated disease progression and death (49). It has also been shown that eGFR values were significantly correlated with COVID-19-related kidney injury, and eGFR values < 60 ml/min/1.73 m^2^ were independently associated with in-hospital mortality (50).

In our previous study, we assessed renal function, according to eGFR values, in patients admitted to the hospital due to COVID-19 (51). Mortality during hospitalization and after 90 and 180 days was significantly higher in the patients with an eGFR < 60 ml/min/1.73 m^2^ (Group B) compared to patients with eGFR values ≥ 60 ml/min/1.73 m^2^ (Group A) (p < 0.001) (51). Mortality in Group B patients was associated with comorbidities, immune impairment, and the frequent development of acute kidney injury. This study showed that the baseline eGFR values determine the course of COVID-19 (51). Our current study also showed that vitamin D concentrations are a significant risk factor for mortality and COVID-19, and patients with lower vitamin D concentrations have a worse prognosis.

D’Avolio et al. (24) stated that patients with COVID-19 had significantly decreased 25(OH)D concentration compared to healthy people (11.1 ng/ml vs. 24.6 ng/ml; p = 0.004). In a study by Meltzer et al. (52), a multivariate analysis showed that a positive test result for COVID-19 was associated with probable vitamin D deficiency status, compared to probable vitamin D sufficiency status (risk ratio [RR] 1.77; 95% CI = 1.12-2.81; p = 0.02). On the other hand, Annweiler et al. (53) found that vitamin D3 supplementation was linked to better three-month survival in elderly patients with COVID-19. Finally, based on systematic reviews and meta-analyses, low vitamin D concentrations were related to a higher risk, severity, and mortality from SARS-CoV-2 infection in most studies (54, 55). Thus, taking into account all the references presented, it seems reasonable to increase blood 25(OH)D concentration as a therapeutic element in patients with COVID-19 (56, 57).

How vitamin D interferes with the outcomes of COVID-19 remains unknown (44). The protective effects of vitamin D in SARS-CoV-2 infection may be mediated through the production of antimicrobial peptides such as cathelicidin and defensin, respiratory barriers, reduced inflammation through tolerogenic effects, and the induction of T-regulatory cells and IL-10, inhibition of IL-12, gamma interferon (IFN-γ), TNF-α, IL-2, and IL-17, along with the modulation of the renin-angiotensin pathway and angiotensin-converting enzyme 2 (ACE-2) downregulation (44, 58–60).

According to current knowledge, it cannot be stated unequivocally for many diseases, disorders, and consequences that vitamin D deficiency is their direct cause (4). The reverse causality hypothesis is gaining traction in light of the results of systematic reviews, meta-analyses, and randomized controlled trials (RCTs) (61, 62). In a review of meta-analyses and RCTs, the authors supported the hypothesis that lower vitamin D concentrations are a consequence of poor health rather than a cause of it (61, 62). In addition, recent research indicates that vitamin D supplementation may prevent frequent upper RTI and asthma exacerbation (62).

The dose of vitamin D has to be appropriately selected depending on the subjects’ baseline 25(OH)D concentration. In a normal-weight adult with a 25(OH)D concentration of 20 ng/ml, 100 IU of vitamin D is needed to increase blood 25(OH)D concentrations by approximately 0.6-1 ng/ml (1). This explains why giving an adult, whose blood 25(OH)D level is 18-20 ng/ml, 1,000 IU of vitamin D is not effective in achieving a blood 25(OH)D of more than 30 ng/ml (1). Central European guidelines consider a vitamin D concentration of 30 to 50 ng/ml as optimal for all potential health advantages (63). On the other hand, the Vitamin D recommendations of the Endocrine Society’s Practice Guidelines refer to 25(OH)D ranging from 40 to 60 ng/ml (64). The authors believe that there should be greater clinical awareness in recognizing specific populations with COVID-19 that require vitamin D supplementation over and above the recommended dose (56, 57). In patients diagnosed with SARS-CoV-2, the daily dose should be higher than those recommended for the elderly, those who are obese, and those with comorbidities. Most of the study results show that vitamin D supplementation would have clinical benefits for patients with COVID-19 in terms of prevention and treatment, including length of hospital stay, mortality, and prognosis recovery (65).

This study was large, homogenous, and only included those with a PCR-confirmed COVID-19 diagnosis who were treated in one center according to the same rules, which may increase the strength of the obtained results.

In this study, some limitations occurred. As a retrospective study, the causality cannot be inferred. Moreover, vitamin D levels were monitored while patients were hospitalized due to SARS-CoV-2 infection. As such, the findings may have been due to reverse causality, which cannot be excluded. SARS-CoV-2 infection may have led to a decreased 25(OH)D concentration, where an enhancement of 25(OH)D1-alpha-hydroxylase enzyme activity due to the systemic inflammatory response associated with COVID-19 can be considered (28). The study did not collect data on vitamin D supplementation or dietary intake.

## 5 Conclusions

The findings indicate an association between SARS-CoV-2 infected patients’ vitamin D concentration and the final course of hospitalization and risk of death. Based on the results of our research, it seems that low vitamin D concentrations should be considered a risk factor for poor prognosis in SARS-CoV-2 infection. From the point of view of public health, it seems important to monitor the concentration of vitamin D, in particular in the elderly and people with heart diseases, and to take preventive measures to achieve the optimal concentrations of vitamin D. Nevertheless, the importance of vitamin D in susceptibility to infection and disease course requires further research.

## Data availability statement

The raw data supporting the conclusions of this article will be made available by the authors, without undue reservation.

## Ethics statement

The studies involving humans were approved by The Institutional Review Board and Bioethics Committee of Wroclaw Medical University, Poland (No: KB-444/2021). The studies were conducted in accordance with the local legislation and institutional requirements. Written informed consent for participation was not required from the participants or the participants' legal guardians/next of kin because The data were collected retrospectively, and written informed consent to participate in the study was not required. The Bioethics Committee approved the publication of anonymized data.

## Author contributions

KK-P and KKo contributed to the conception and design of the study. KK-P, KKo, and KKu contributed to the methodology of the study. KKu performed the statistical analysis. KKo, KK-P, AM-W, KKu, BA, AD, KKa, MPo, MPr, JS, KM, and EJ conducted research, investigation process, and data curation of the study. KKo and KK-P wrote the first draft of the manuscript. KKu and AM-W wrote sections of the manuscript. KM and EJ supervised the study. All authors contributed to the article and approved the submitted version.
